# Development and validation of an immune checkpoint-based signature to predict prognosis in nasopharyngeal carcinoma using computational pathology analysis

**DOI:** 10.1186/s40425-019-0752-4

**Published:** 2019-11-13

**Authors:** Ya-Qin Wang, Yu Zhang, Wei Jiang, Yu-Pei Chen, Shuo-Yu Xu, Na Liu, Yin Zhao, Li Li, Yuan Lei, Xiao-Hong Hong, Ye-Lin Liang, Jun-Yan Li, Lu-Lu Zhang, Jing-Ping Yun, Ying Sun, Ying-Qin Li, Jun Ma

**Affiliations:** 1 Department of Radiation Oncology, State Key Laboratory of Oncology in South China, Collaborative Innovation Center of Cancer Medicine, Guangdong Key Laboratory of Nasopharyngeal Carcinoma Diagnosis and Therapy, Sun Yat-sen University Cancer Center, Guangzhou, 510060 People’s Republic of China; 2 Department of Pathology, Sun Yat-sen University Cancer Center, State Key Laboratory of Oncology in South China, Collaborative Innovation Center of Cancer Medicine, Guangzhou, 510060 Guangdong China; 3 Department of Radiation Oncology, Affiliated Hospital of Guilin Medical University, 15 Lequn Road, Guilin, 541001 China; 4 Department of Medical Imaging, Sun Yat-sen University Cancer Center, State Key Laboratory of Oncology in South China, Collaborative Innovation Center of Cancer Medicine, Guangzhou, 510060 Guangdong China; 5 Department of Molecular Diagnostics, Sun Yat-sen University Cancer Center, State Key Laboratory of Oncology in South China, Collaborative Innovation Center of Cancer Medicine, Guangzhou, 510060 Guangdong China

**Keywords:** Immune checkpoint-based signature, Nasopharyngeal carcinoma, Computational pathology analysis, Tumour-immune microenvironment, EBV-DNA

## Abstract

**Background:**

Immunotherapy, especially immune checkpoint inhibition, has provided powerful tools against cancer. We aimed to detect the expression of common immune checkpoints and evaluate their prognostic values in nasopharyngeal carcinoma (NPC).

**Methods:**

The expression of 9 immune checkpoints consistent with 13 features was detected in the training cohort (*n* = 208) by immunohistochemistry and quantified by computational pathology. Then, the LASSO cox regression model was used to construct an immune checkpoint-based signature (ICS), which was validated in a validation cohort containing 125 patients.

**Results:**

High positive expression of PD-L1 and B7-H4 was observed in tumour cells (TCs), whereas PD-L1, B7-H3, B7-H4, IDO-1, VISTA, ICOS and OX40 were highly expressed in tumour-associated immune cells (TAICs). Eight of the 13 immune features were associated with patient overall survival, and an ICS classifier consisting of 5 features (B7-H3TAIC, IDO-1TAIC, VISTATAIC, ICOSTAIC, and LAG3TAIC) was established. Patients with high-risk scores in the training cohort had shorter overall (*P* < 0.001), disease-free (*P* = 0.002), and distant metastasis-free survival (*P* = 0.004), which were confirmed in the validation cohort. Multivariate analysis revealed that the ICS classifier was an independent prognostic factor. A combination of the ICS classifier and TNM stage had better prognostic value than the TNM stage alone. In addition, the ICS classifier was significantly associated with survivals in patients with high EBV-DNA load.

**Conclusions:**

We determined the expression status of nine immune checkpoints consistent with 13 features in NPC and further constructed an ICS prognostic model, which might add prognostic value to the TNM staging system.

## Background

Nasopharyngeal carcinoma (NPC) is prevalent in southern China, Southeast Asia, North Africa, the Middle East and Alaska [1]. With the advent of intensity-modulated radiotherapy and combined chemoradiotherapy, the local control rate has been significantly improved, and distant metastasis has become the main cause of death in NPC, making it urgent to seek novel effective treatment methods [2]. NPC is characterized by prevailing Epstein-Barr virus (EBV) infection and a heavy infiltration of immune cells around tumour lesions [3, 4]. Recent studies showed that increased TILs (tumor-infiltrating lymphocytes) and CD3+ T cells (total T cells) was associated with improved survival for NPC patients [4, 5]. However, cancer cell could still keep growing in the patients with high infiltration of lymphocytes, which suggested the existence of immunosuppressive microenvironment in NPC patients [6, 7]. Due to the efficacy of improving immunosuppressive microenvironment, immunotherapy was suggested to be a promising therapeutic method for NPC patients.

Accumulating studies report that the immunosuppressive tumour microenvironment makes immune cells exhausted and anergic, eventually enabling cancer cells to evade host immune-mediated elimination [6]. Immune checkpoints expressed on tumour or inflammatory cells play vital roles in inhibiting or enhancing the anti-tumour immune response, and blocking inhibitory immune checkpoints has become an attractive anti-tumour strategy [8, 9]. Actually, several important single-arm trials evaluating monoclonal antibodies against programmed cell death protein 1 (PD-1) in recurrent or metastatic nasopharyngeal carcinoma have been reported, where PD-1 inhibitors is effective in only 20~30% of NPC patients [10, 11]. Those indicated that the tumour microenvironment is intricate and other immune checkpoints, such as B7-H3, LAG3, and VISTA, might exist. However, the expression levels of most immune checkpoints in NPC are still unknown, and there is a need to systematically evaluate the expression statuses of all immune checkpoints in NPC.

In this study, based on computational pathology analysis, we simultaneously detected the expression status of nine immune checkpoints consistent with 13 features and evaluated the comprehensive immunosuppressive status of the NPC microenvironment. We then explored the prognostic values of these immune checkpoint features and developed an immune checkpoint-based signature (ICS) to predict the clinical outcomes of NPC patients, which could divide patients into different risk subgroups and might add prognostic value to the TNM staging system.

## Methods

### Clinical specimens

We retrospectively collected 333 paraffin-embedded NPC specimens for this study. A total of 208 specimens obtained at the Sun Yat-sen University Cancer Center (Guangzhou, China) between January 2011 and December 2013 were designated as the training cohort, while 125 samples obtained at the Affiliated Hospital of Guilin Medical University (Guilin, China) between January 2010 and June 2014 were designated as the validation cohort. All patients from the Guangzhou cohort underwent intensity-modulated radiation therapy (IMRT), and all patients from the Guilin cohort underwent two-dimensional radiotherapy (2D-RT). No patients had received any antitumour therapy before biopsy sampling, and all of the patients were pathologically diagnosed with NPC. All patients were restaged according to the 8th AJCC TNM staging system [12]. This study was approved by the Institutional Ethical Review Boards of both hospitals, and written informed consent was obtained from each patient. This study is reported according to the REMARK criteria [13].

### Immunohistochemistry (IHC)

Based on previous studies [14–17], we selected 9 prognostic immune checkpoints for IHC staining: PD-L1, B7-H3, B7-H4, IDO-1, LAG-3, VISTA, TIM-3, ICOS and OX40. IHC was performed as previously described [18]. The following primary antibodies were used: anti-PD-L1 (clone E1L3N, 1:400 dilution; Cell Signaling Technology, CST, Beverly, Massachusetts), anti-B7-H3 (clone D9M2L, 1:400; CST), anti-B7-H4 (clone HPA054200, 1:800; Sigma-Aldrich, Ronkonkoma, NY, USA), anti-IDO-1 (clone D5J4E; 1:800; CST), anti-LAG3 (clone D2G40, 1:100; CST), anti-VISTA (clone D1L2G, 1:800; CST), anti-TIM3 (clone D5D5R, 1:400; CST), anti-ICOS (clone D1K2T, 1:1600; CST), and anti-OX40 (ab119904, 1:1600; Abcam, Cambridge, UK).

### Computational pathology analysis

A full view of each IHC slide was digitally scanned using a ScanScope Aperio AT2 slide scanner (Leica Microsystems) at 400× magnification. All images were auto-examined using computational pathology analysis, and the expression was quantified as the percentage of tumour cells (TCs) or tumour-associated immune cells (TAICs) expressing the immune checkpoints. As the immune checkpoints PD-L1, B7-H3, B7-H4, and IDO-1 are expressed by both TCs and TAICs, these checkpoints were assessed in both compartments. In contrast, given their predominant expression in TAICs, LAG3, VISTA, TIM3, ICOS, and OX40 were assessed only in the tumour stroma compartment (Additional file 1: Figure S1). In total, there are 13 features.

**Fig. 1:**
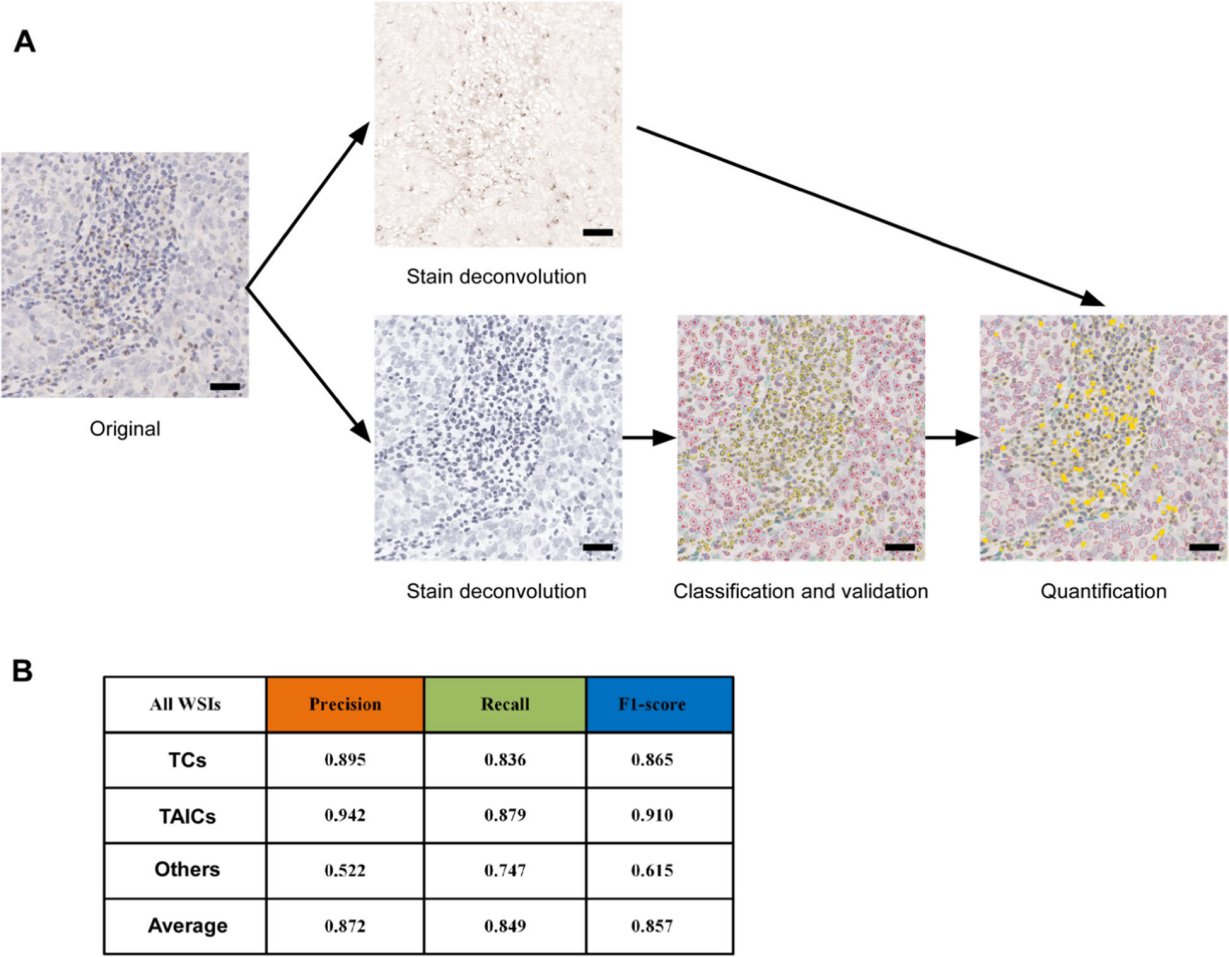
Computational pathology analysis. **a** Histology image analysis pipeline and validation; (**b**) Precision, recall and F1-score for each of the three cell classes. The scale bar represents 30 μm

In brief, the computational pathology analysis consisted of five stages: 1) manual annotation of the individual cell nuclei into TCsand TAICs by two pathologists; 2) stain deconvolution of the IHC staining from the haematoxylin counterstaining; 3) automated segmentation of the nuclei in the haematoxylin channel; 4) automated classification of the cells into TCs, TAICs and other cells using the Xception deep learning model [19]; and 5) quantification of the positive cell percentage for each immune checkpoint (Fig. 1a). Detailed descriptions of the computational pathology analysis are provided in the supplementary materials. Computational pathology analysis showed a high consistency with pathological classification, with an accuracy rate of 83.6% for TC identification and 87.9% for TAIC identification (Fig. 1b).

### Construction of the ICS

We adopted a penalized Cox regression model to select the most useful prognostic features out of all 13 immune checkpoint features [20] and then constructed an ICS for predicting survival in the training cohort. The “glmnet” package was used to perform a least absolute shrinkage and selection operator (LASSO) Cox regression model analysis. Ten-time cross validations with the Lambda.min criteria were used to determine the optimal values of λ, and a value of λ = 0.038 with log (λ) = − 3.269 was chosen. Based on this value, IDO1_TAIC,_ VISTA _TAIC_, B7-H3 _TAIC_, ICOS _TAIC_ and LAG3 _TAIC_ were selected to construct the prediction model with the coefficients weighted by the penalized Cox model in the training cohort. We then used X-tile software (version 3.6.1; Yale University, New Haven, CT, USA) to generate the optimal cutoff values for the ICS scores based on the associations with patient overall survival (OS) [21]. The thresholds for the scores that were produced by the predictive model were used to separate patients into low-risk and high-risk groups.

### DNA extraction and real-time QuantitativePolymerase chain reaction

The plasmatic EBV DNA concentrations were routinely measured before treatment using quantitative polymerase chain reaction, as described in the Additional file 1 [22]. A cut-off level of 2000 copies/mL was chosen to define low and high pretreatment EBV DNA levels [23, 24].

### Statistical analyses

Our primary endpoint was OS, and secondary endpoints included disease-free survival (DFS) and distant metastasis-free survival (DMFS). We calculated OS from the first day of treatment to the date of death from any cause, DFS from the first day of treatment to the date of the first relapse at any site or death from any cause (whichever occurred first) and DMFS from the first day of treatment to the first distant relapse.

The associations between the ICS and clinicopathological variables were calculated using the χ2 test or Fisher’s exact test. Receiver operating characteristic (ROC) curve analysis was used to determine optimal cutoff values separating high and low expression for the 13 immune checkpoint features in the training cohort with respect to OS. The Kaplan-Meier method was used to estimate OS, DFS and DMFS, the log-rank test was used to compare differences, and hazard ratios (HRs) were calculated using univariate Cox regression analysis. Multivariate Cox regression analysis with backward selection was used to test the independent significance of different factors. Significant variables (*P* ≤ 0.1) were included in the multivariate analysis, and only independent prognostic factors were retained in the multivariate model. In addition, we established a prognostic score model combining the ICS and TNM stage [25, 26]. ROC curves were used to compare its prognostic validity with the TNM stage or ICS alone models. We also did subgroup analysis according to the pre-treatment plasma EBV-DNA levels.

All statistical tests were two-sided and considered significant when the *p* value was less than 0.05. Statistical analyses were performed using Statistical Package for the Social Sciences (SPSS) v22.0 (IBM, Armonk, NY, USA) and R software (R version 3.2.3; rms package, “rpart” package version 4.1–10, http://www.r-project.org/; “glmnet” package). The authenticity of this article has been validated by uploading the key raw data onto the Research Data Deposit public platform (http://www.researchdata.org.cn), with the approval RDD number as RDDB2019000556.

## Results

### Patient characteristics and immune checkpoint expression

We collected 333 pretreatment, non-metastatic NPC specimens that were obtained at two academic institutions for this study. Additional file 2: Table S1 shows the clinicopathological characteristics of the patients in the training cohort (*n* = 208) or validation cohort (*n* = 125). All patients received radiotherapy, and 307 (92.2%) patients received platinum-based chemotherapy. The median follow-up time was 69.7 months (interquartile range (IQR) 65.1–72.8) for the patients in the training cohort and 58 months (IQR 41–69) for those in the validation cohort.

Representative images of immunohistochemical staining for the 9 immune checkpoints consistent with 13 features tested are shown in Additional file 1: Figure S1. Based on computational pathology analysis, the expression of the immune checkpoints was digitally measured and quantified as the positive expression percentages of TCs and TAICs. Using four cutoff values (> 1, > 5, > 25, and >  50%), which have been frequently used in reports on the expression of PD-L1, we determined the distribution of NPC patients expressing the immune checkpoints in the training cohort. In addition, the median percentages of all immune checkpoints were also determined. With a median percentage greater than 10%, high positive expression of PD-L1 and B7-H4 was observed in TCs, whereas all immune checkpoints except for LAG3 and TIM3 were highly expressed in TAICs (Table 1). In addition, we analysed the co-expression status of four immune checkpoints in TCs and found that PD-L1, B7-H4, and IDO-1 expression was the most common combination of simultaneously expressed markers, as this pattern was seen in 16% of the NPC specimens (Additional file 2: Table S2).

**Table 1:** Expression levels of 13 features regarding 9 immune checkpoint markers in nasopharyngeal carcinoma

No. of NPC Patients (%) with Expression Above the Cutoff Value					
Marker	Median	> 1%	> 5%	> 25%	> 50%
TAIC expression					
PD-L1	12.97	201 (97)	151 (73)	59 (28)	16 (8)
B7-H3	26.13	196 (94)	175 (84)	105 (50)	50 (24)
B7-H4	22.06	205 (99)	195 (94)	90 (43)	21 (10)
IDO-1	20.75	206 (99)	187 (90)	83 (40)	14 (7)
LAG3	5.55	197 (95)	109 (52)	15 (7)	5 (2)
VISTA	12.46	205 (99)	169 (81)	30 (14)	3 (1)
TIM3	2.75	183 (88)	62 (30)	0 (0)	0 (0)
ICOS	26.21	207 (100)	204 (98)	111 (53)	9 (4)
OX40	19.95	208 (100)	192 (92)	80 (38)	24 (12)
TC expression					
PD-L1	10.44	191 (92)	136 (65)	49 (24)	22 (11)
B7-H3	7.01	165 (79)	115 (55)	50 (24)	19 (9)
B7-H4	13.41	200 (96)	162 (78)	57 (27)	13 (6)
IDO-1	7.66	195 (94)	131 (63)	23 (11)	6 (3)

Abbreviations: *TC* tumour cell, *TAIC* tumour-associated immune cell

### Prognostic value of immune checkpoint expression

**Fig. 2:**
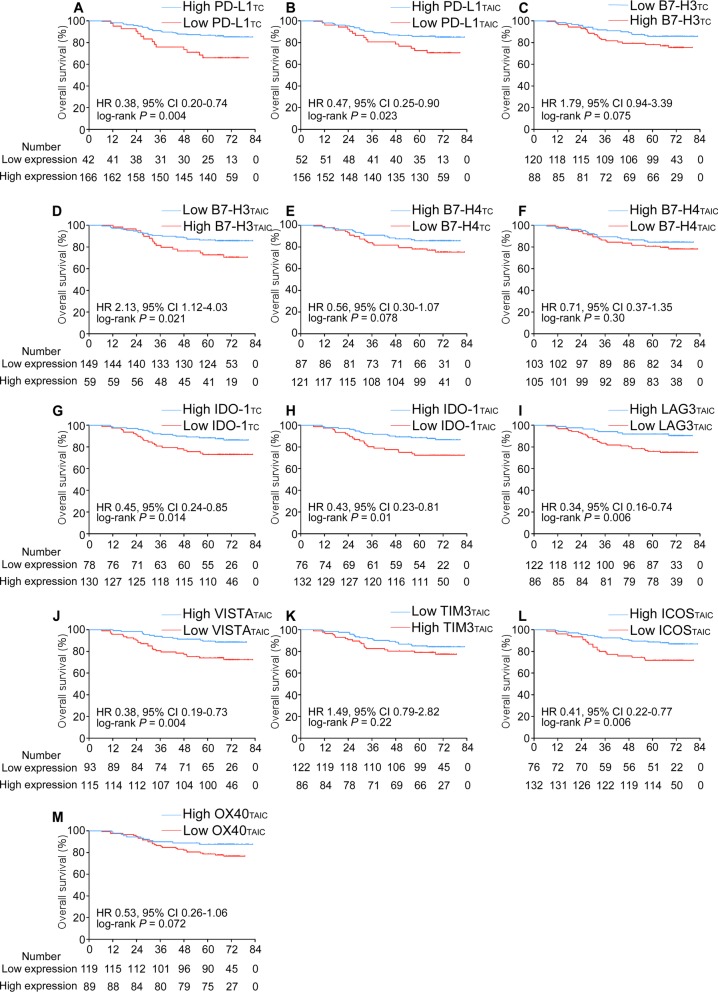
Kaplan-Meier curves for overall survival according to the 13 immune checkpoint features. Plots show (**a**) PD-L1_TC_; (**b**) PD-L1_TAIC_; (**c**) B7-H3_TC_; (**d**) B7-H3_TAIC_; (**e**) B7-H4_TC_; (**f**) B7-H4_TAIC_; (**g**) IDO-1_TC_; (**h**) IDO-1_TAIC_; (**i**) LAG3_TAIC_; (**j**) VISTA_TAIC_; (**k**) TIM-3_TAIC_; (**l**) ICOS_TAIC_ and (**m**) OX40_TAIC_ in the training cohort. Abbreviations: TC, tumour cell; TAIC, tumour-associated immune cell; HR, hazard ratio; and CI, confidence interval

Furthermore, we explored the prognostic value of the 13 immune checkpoint features in the training cohort. As shown in Fig. 2, eight of the features were significantly associated with patient survival. The patients with high expression of PD-L1 in either their TCs (HR 0.38, 95% confidence interval (CI) 0.20–0.74, *P* = 0.004) or TAICs (HR 0.47, 95% CI 0.25–0.90, *P* = 0.023) had better OS than the patients with low expression of PD-L1. Similar results were observed for IDO-1 expression in both the TCs (HR 0.45, 95% CI 0.24–0.85, *P* = 0.014) and TAICs (HR 0.43, 95% CI 0.23–0.81, *P* = 0.01). In addition, high expression of LAG3 (HR 0.34, 95% CI 0.16–0.74, *P* = 0.006), VISTA (HR 0.38, 95% CI 0.19–0.73, *P* = 0.004), or ICOS (HR 0.41, 95% CI 0.22–0.77, *P* = 0.006) in the TAICs was associated with better OS than low expression, while high expression of B7-H3 in the TAICs (HR 2.13, 95% CI 1.12–4.03, *P* = 0.021) was associated with poorer OS than low expression (Fig. 2). The associations between the 13 immune checkpoint features and DFS or DMFS are listed in Additional file 1: Figure S2 and Figure S3.

### ICS construction and its association with prognosis

To construct an immune checkpoint-based prognostic model, we identified 5 immune checkpoint features that were significantly associated with OS in the training cohort using penalized LASSO Cox regression models (Additional file 1: Figure S4). Then, a risk score was calculated for each patient using a formula that included 5 features weighted by their regression coefficient: Risk score = (0.6995× positive percentage of B7-H3_TAIC_) - (0.0054× positive percentage of IDO-1_TAIC_) - (0.4039× positive percentage of VISTA_TAIC_) - (1.6908× positive percentage of ICOS_TAIC_) - (0.0710× positive percentage of LAG3_TAIC_).

**Fig. 3:**
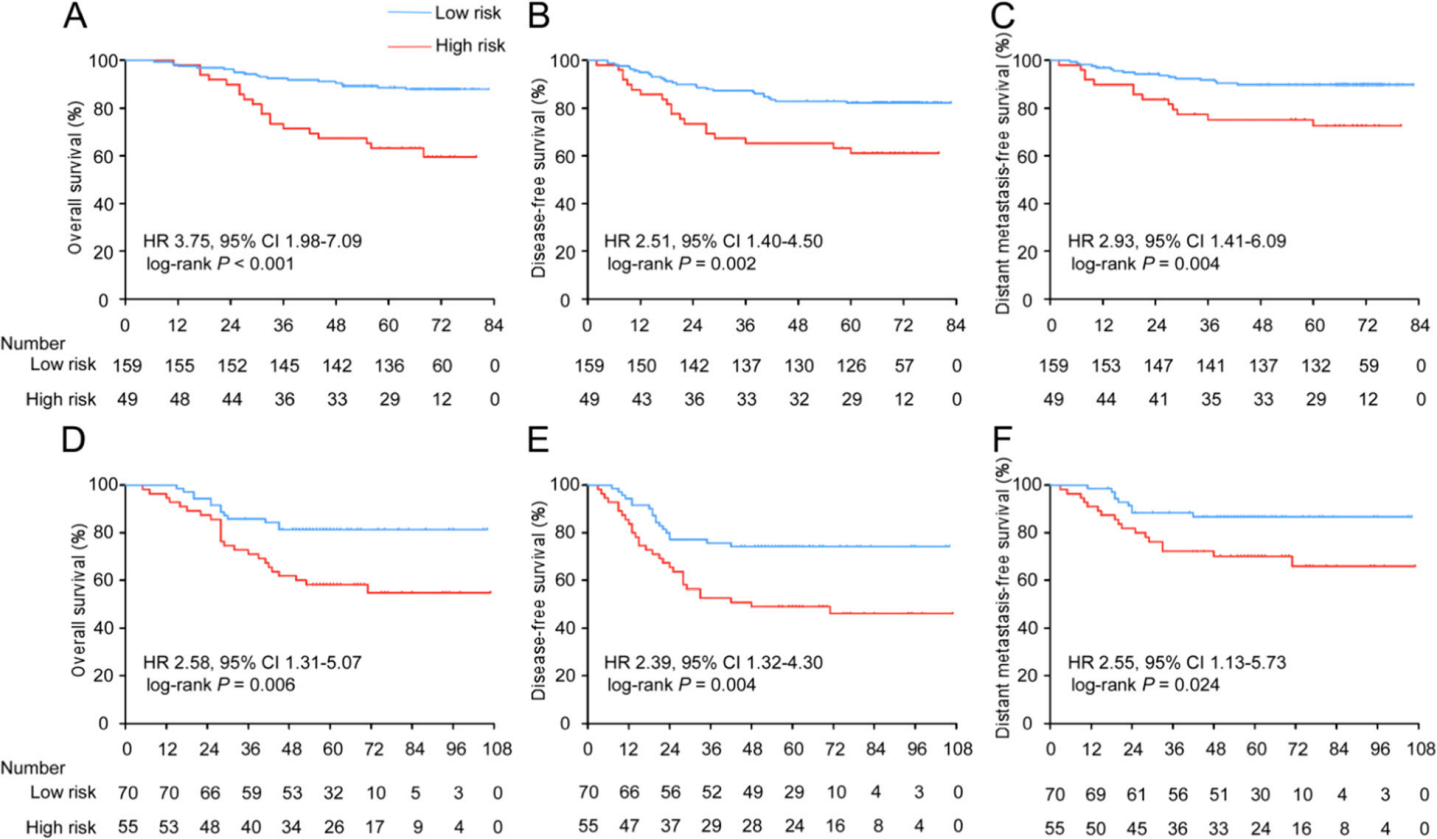
Kaplan-Meier curves for overall, disease-free and distant metastasis-free survival according to the ICS. Plots show (**a**) overall survival, (**b**) disease-free survival and (**c**) distant metastasis-free survival in the training cohort and (**d**) overall survival, (**e**) disease-free survival and (**f**) distant metastasis-free survival in the validation cohort. Abbreviations: ICS, immune checkpoint-based signature; HR, hazard ratio; and CI, confidence interval

After using X-tile plots to generate the optimal cutoff value (− 0.16) for the risk score (Additional file 1: Figure S5), we assigned 159 patients in the training cohort into the low-risk group and 49 patients into the high-risk group. The high-risk group had a shorter 5-year OS rate than the low-risk group (61.2% vs. 88.1%, respectively, HR 3.75, 95% CI 1.98–7.09, *P* < 0.001). The patients with high-risk scores also had shorter DFS (HR 2.51, 95% CI 1.40–4.50, *P* = 0.002) and DMFS (HR 2.93, 95% CI 1.41–6.09, *P* = 0.004) than the patients with low-risk scores (Fig. 3a-c).

### Validation of the prognostic value of the ICS

To validate whether the ICS has similar prognostic value in different populations, we tested the 5 immune checkpoint features in a validation cohort of 125 NPC patients and then used the formula and cutoff point developed from the training cohort to stratify the patients into low-risk (*n* = 70) and high-risk (*n* = 55) groups. The patients with high-risk scores had shorter OS (56.4% vs. 81.4%, respectively, HR 2.58, 95% CI 1.31–5.07, *P* = 0.006), DFS (HR 2.39, 95% CI 1.32–4.30, *P* = 0.004) and DMFS (HR 2.55, 95% CI 1.13–5.73, *P* = 0.024; Fig. 3d-f) than those with low-risk scores. The 5-year OS, DFS, and DMFS rates in each ICS group and the number of patients who had an event in each risk group are listed in Additional file 2: Table S3 and Table S4, respectively.

We performed univariate analyses with the training and validation cohorts, and Additional file 2: Table S5, Table S6 and Table S7 shows the associations among the ICS, clinicopathological characteristics and patient clinical outcomes. The ICS was significantly associated with OS, DFS, and DMFS in the two cohorts. Multivariate Cox regression analysis showed that the ICS remained a powerful and independent prognostic factor for OS, DFS, and DMFS in the training cohort (OS: HR 3.62, 95% CI 1.91–6.87, *P* < 0.001; DFS: HR 2.43, 95% CI 1.35–4.35, *P* = 0.003; and DMFS: HR 2.77, 95% CI 1.33–5.77, *P* = 0.007) as well as in the validation cohort (OS: HR 2.59, 95% CI 1.32–5.10, *P* = 0.006; DFS: HR 2.38, 95% CI 1.32–4.30, *P* = 0.004; and DMFS: HR 2.55, 95% CI 1.13–5.72, *P* = 0.024). In addition, the TNM stage and EBV-DNA levels were also significantly associated with OS, DFS and DMFS in the multivariate analysis (Additional file 2: Table S8).

### Prognostic score model combined the ICS and TNM stage

TNM stage is the determinant for predicting prognosis and guiding treatment currently, but its accuracy is limited as it based on anatomical information and it need to be supplement with molecular indicators.

**Fig. 4:**
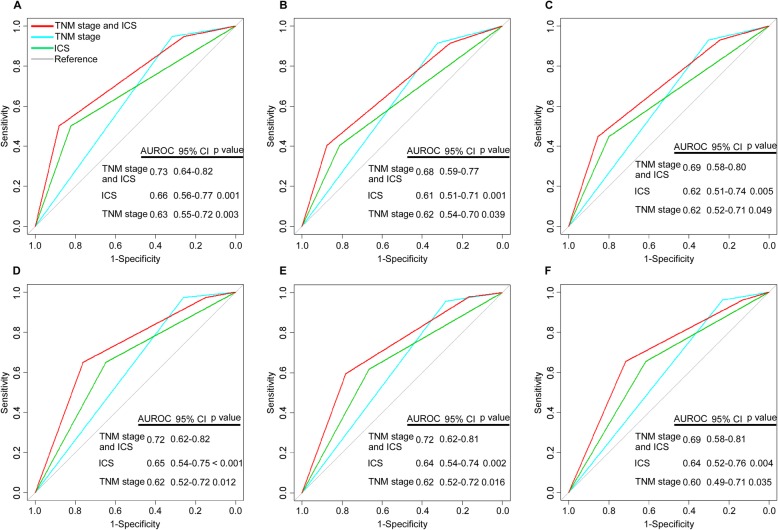
Comparisons of the sensitivity and specificity for the prediction of overall, disease-free and distant metastasis-free survival by the combined ICS and TNM stage model, the TNM stage alone model, and the ICS alone model. Receiver operating characteristics (ROC) curves of (**a**) overall survival, (**b**) disease-free survival and (**c**) distant metastasis-free survival in the training cohort and (**d**) overall survival, (**e**) disease-free survival and (**f**) distant metastasis-free survival in the validation cohort. *P* values show the area under the ROC (AUROC) of the combined ICS and TNM stage model versus AUROCs of the TNM stage alone model or the ICS alone model

To develop a more sensitive model to predict prognosis of NPC patients, we established a prognostic score model combining the ICS and TNM stage based on the multivariate Cox regression analysis. The regression coefficient of the ICS was divided by the regression coefficient of the TNM stage, and then rounded into an integer value to generate the risk score (Additional file 2: Table S9). We calculated each patient a cumulative risk score, and used ROC analysis to compare the sensitivity and specificity of the prognostic score model with the TNM stage or ICS alone model. Combination of the ICS and TNM stage showed significantly better prognostic value than the TNM stage alone for OS (area under ROC (AUROC) 0.73 [95% CI 0.64–0.82] vs 0.63 [0.55–0.72]; *P* = 0.003), DFS (0.68 [95% CI 0.59–0.77] vs 0.62 [0.54–0.70]; *P* = 0.039), and DMFS (0.69 [95% CI 0.58–0.80] vs 0.62 [0.52–0.71]; *P* = 0.049) in the training cohort, which were confirmed in the validation cohort (OS, 0.72 [95% CI 0.62–0.82] vs 0.62 [0.52–0.72]; *P* = 0.012; DFS, 0.72 [95% CI 0.62–0.81] vs 0.62 [0.52–0.72]; *P* = 0.016; DMFS, 0.69 [95% CI 0.58–0.81] vs 0.60 [0.49–0.71]; *P* = 0.035) (Fig. 4).

### Association between the ICS and EBV-DNA levels

**Fig. 5:**
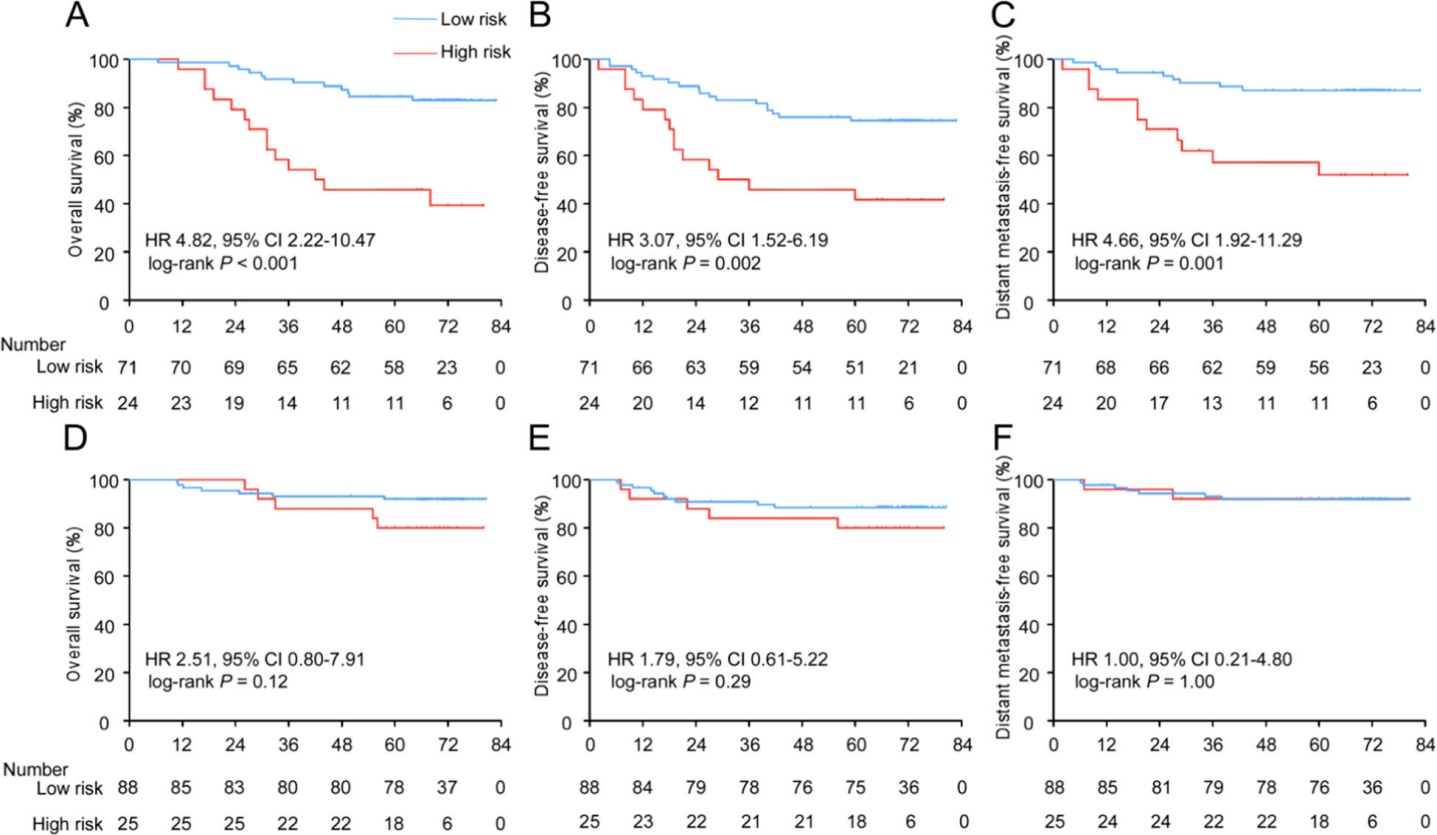
Kaplan-Meier curves for overall, disease-free and distant metastasis-free survival of patients grouped by their EBV-DNA level and then stratified according to the ICS. Plots show (**a**) overall survival, (**b**) disease-free survival and (**c**) distant metastasis-free survival in the EBV-DNA level > 2000 copy/mL subgroup and (**d**) overall survival, (**e**) disease-free survival and (**f**) distant metastasis-free survival in the EBV-DNA level ≤ 2000 copy/mL subgroup. Abbreviations: ICS, immune checkpoint signature; HR, hazard ratio; and CI, confidence interval

NPC is closely associated with EBV infection, which has been reported to be involved in the regulation of immune-inhibitory biomolecules [27]. We analysed whether the EBV-DNA burden could affect the predictive efficacy of the ICS in 208 NPC patients from the Guangzhou training cohort. After the patients were divided into different subgroups by their pretreatment plasma EBV-DNA level, Kaplan-Meier curves showed that stratification by the ICS resulted in significant differences in OS (HR 4.82, 95% CI 2.22–10.47, *P* < 0.001), DFS (HR 3.07, 95% CI 1.52–6.19, *P* = 0.002), and DMFS (HR 4.66, 95% CI 1.92–11.29, *P* = 0.001) in the patients with an EBV-DNA level > 2000 copy/mL (Fig. 5a-c). However, in the patients with an EBV-DNA level ≤ 2000 copy/mL, we did not find a significant association between the ICS and any of the outcomes (Fig. 5d-f). The 5-year OS, DFS, and DMFS rates in each risk group and the number of patients who had an event in each risk group among the different EBV-DNA burden groups are listed in Additional file 2: Table S3 and Table S4.

## Discussion

In this study, we determined the expression of 13 immunologic variables derived from 9 immune checkpoints and evaluated their prognostic value in NPC patients. Furthermore, we developed and validated a novel prognostic model (ICS) based on the expression of 5 immune checkpoint features, which could improve the ability to predict the clinical outcome of NPC patients when combined with the TNM stage, especially that of patients with a high pre-treatment EBV-DNA burden. In addition, based on anatomical information, TNM staging is an important factor in predicting prognosis. Conversely, the ICS signature could provide the immune microenvironment status of nasopharyngeal carcinoma and may add prognostic value to the TNM staging system. We developed a prognostic score model combining ICS and TNM stage had better prognostic value than did TNM stage alone in the training cohort and the validation cohort. The prognostic score model allows for a more accurate classification of NPC patients at different risks. To our knowledge, this is the first study to simultaneously measure 13 different immunologic variables derived from 9 immune checkpoints in the tumour microenvironment using digital computational analysis and to construct an immune-related prognostic model for NPC.

Immune escape is a hallmark of tumour progression [6]. Important studies have found that immune-inhibitory and immune-activating molecules expressed on TCs or TAICs are involved in the regulation of tumour immune escape [14]. These molecules have been found to be abnormally expressed in a variety of cancers and associated with patient prognosis [15, 28]. Furthermore, some of these immune checkpoints have been shown to be promising treatment targets [14, 28]. However, the expression of these immune checkpoints in the tumour-immune microenvironment of NPC is still unclear. In this study, the expression of 9 immune modulators (out of a total of 13 immune features) by TCs or TAICs was automatically quantified, and a 5-immune feature-based classifier was established to predict the survival of NPC patients, and these predictions were validated in an external cohort. Importantly, our results demonstrated that ICS was an independent prognostic factor in patients receiving either IMRT (SYSUCC cohort) or 2D-RT (Guilin cohort). Therefore, ICS is a promising prognostic classifier, which could be widely used to predict the prognosis of NPC patients regardless of RT techniques used. In addition, a prognostic score model combined the ICS classifier and TNM stage was constructed and had a better prognostic value than the TNM stage alone, which could guide a more personalized therapy. Our study of the expression of multiple immune checkpoints can help to understand the immune state of tumours in individuals and potentially improve therapeutic approaches for patients with different immunosuppressive mechanisms.

Computational pathology analysis has been established for several decades [29]. In recent years, it has gained great attention due to the capabilities of whole-slide scanning and accurate large-scale analysis without subjective bias. In addition, emerging biomarker-based patient stratification calls for the accurate quantitative evaluation of molecular properties [30]. Since the structural microscopic morphology of NPC is diverse and complex, report variation exists between different pathologists in identifying the percentages of immune checkpoint-expressing cells among TCs and TAICs. Thus, it is necessary to deeply explore the immune checkpoint properties of NPC using computational pathology analysis. Especially for patients receiving immunotherapy, computational pathology analysis makes immune checkpoint expression evaluation scalable to a large number of image features contained in whole-slide pathology images, and it will hopefully identify new effective biomarkers that can select appropriate patients for immunotherapy. In this study, we quantified 13 immune features derived from 9 immune checkpoints through computational pathology analysis. Our computational pathology analysis, which was developed based on the Xception model, achieved good performance in identifying TC and TAIC nuclei. Moreover, this analysis can obtain a large amount of quantitative information with high speed, which provides an effective prognostic tool for NPC patients.

EBV plays an important role in the pathogenesis of NPC, and the pre-treatment plasma EBV-DNA load correlates with cancer stage and clinical outcome in endemic NPC [3, 23]. Here, we performed a subgroup analysis to explore whether the EBV-DNA load affects the prognostic value of our ICS prognostic model. Our data suggested that the OS, DFS, and DMFS of patients with an EBV-DNA level > 2000 copy/mL were largely governed by the state of the ICS, whereas those of the patients with an EBV-DNA level ≤ 2000 copy/mL were not. Accumulating studies report that EBV DNA might be released by cancer cells during apoptosis, which could reflect the tumor burden of patients [31, 32]. In addition, immunosuppressive microenvironment could facilitate tumor progression [6]. Therefore, we presumed that the existence of immunosuppressive microenvironment in NPC patients might lead to high tumor burden, which released more EBV DNA in the plasma. Moreover, the patients with high-risk scores had shorter DMFS than those with low-risk scores, and there was no significant association between the ICS and LRRFS both in the training and validation cohorts. We speculated that the high-risk score of ICS mainly lead to distant metastasis, and the locoregional control of NPC were regulated by other mechanisms. In this regard, EBV DNA load is positively correlated to the risk of distant metastatization [23]. Consistently, we observed that patients experienced unfavourable DMFS in groups with high EBV DNA load.

Actually, our study has limitation due to the objective reasons. As radiotherapy or combined with chemotherapy is now the standard treatment for locoregionally nasopharyngeal carcinoma, surgery is not recommended [33]. Therefore, the whole tumor could not be obtained from NPC patients. In this study, we evaluated the expression of each immune checkpoint on single biopsy of NPC patients, which might represent local immunity pattern.

In our present study, we systemically evaluated the immunosuppressive status of the NPC tumour-immune microenvironment. We determined the expression statuses and prognostic values of nine immune checkpoints consistent with 13 features in NPC and further constructed an ICS prognostic model based on 5 immune checkpoint features and combined with the TNM stage, which allows for a more accurate classification of patients at different risks.

## Conclusions

We developed and validated an immune checkpoint-based signature consisting of 5 immune checkpoint features to predict clinical outcomes in nasopharyngeal carcinoma (NPC), which allows for a more accurate classification of patients at different risks and might add prognostic value to the TNM staging system.

## Supplementary information

**Additional file 1: Figure S1.** Representative images of immune checkpoint expression in TCs and TAICs from nasopharyngeal carcinoma determined by immunohistochemistry. **Figure S2.** Kaplan-Meier curves for disease-free survival according to 13 immune checkpoint features. **Figure S3.** Kaplan-Meier curves for distant metastasis-free survival according to 13 immune checkpoint features. **Figure S4.** Construction of the ICS, a classifier comprising 5 immune checkpoint features. **Figure S5.** Determination of the optimum cutoff value for the ICS risk score.

**Additional file 2: Table S1.** Clinicopathological characteristics of the patients in the training and validation cohorts stratified according to the ICS. **Table S2.** Immune checkpoint co-expression by tumour cells (TCs) in the training cohort. **Table S3.** Five-year OS, DFS and DMFS estimates for different groups. **Table S4.** Number of events for different groups. **Table S5.** Univariate analysis of factors associated with overall survival in the training and validation cohorts. **Table S6.** Univariate analysis of factors associated with disease-free survival in the training and validation cohorts. **Table S7.** Univariate analysis of factors associated with distant metastasis-free survival in the training and validation cohorts. **Table S8.** Multivariable Cox regression analysis of factors associated with survival in the training and validation cohorts. **Table S9.** Summary of the multivariable analyses of prognostic factors for OS, DFS, DMFS and corresponding risk score in the training set of 208 nasopharyngeal carcinoma patients.

## Data Availability

The datasets used and/or analysed during the current study are available from the corresponding author on reasonable request.

## Abbreviations

CI: Confidence interval
DFS: Disease-free survival
DMFS: Distant metastasis-free survival
HR: Hazard ratio
ICS: Immune checkpoint-based signature
NPC: Nasopharyngeal carcinoma
OS: Overall survival
TAIC: Tumour-associated immune cell
TC: Tumour cell
