# Supplementary material for: Development and validation of an immune checkpoint-based signature to predict prognosis in nasopharyngeal carcinoma using computational pathology analysis
Source: J Immunother Cancer. 2019 Nov 13;7:298. doi: 10.1186/s40425-019-0752-4 (PMC6854706; doi:10.1186/s40425-019-0752-4)
Supplement: Supplementary file 1 — Additional file 1: Figure S1. Representative images of immune checkpoint expression in TCs and TAICs from nasopharyngeal carcinoma determined by immunohistochemistry. Figure S2. Kaplan-Meier curves for disease-free survival according to 13 immune checkpoint features. Figure S3. Kaplan-Meier curves for distant metastasis-free survival according to 13 immune checkpoint features. Figure S4. Construction of the ICS, a classifier comprising 5 immune checkpoint features. Figure S5. Determination of the optimum cutoff value for the ICS risk score. [file 40425_2019_752_MOESM1_ESM.docx]

**Supplementary Methods**

**Computational pathology analysis**

Stain deconvolution [1] was first performed on all IHC-stained images to separate IHC staining from haematoxylin counterstaining. Each nucleus in the haematoxylin channel was then segmented using a regional convolutional neural network (R-CNN) [2] trained on thirty images from the public dataset MICCAI Monuseg [3]. This dataset has various types of tissue annotated by experienced pathologists.

To identify tumour cells (TCs) and tumour-associated immune cells (TAICs), we first established a dataset by randomly selecting five fields of view (1024*1024 pixels) from whole-slide images (WSIs) of each IHC-stained slide and then had two pathologists who were blinded to the image analysis results and clinical outcomes manually annotate each nucleus as a TC or TAIC. TCs were annotated with red dots, and TAICs were annotated with yellow dots (Fig. 1A). If the cell was neither a TC nor a TAIC, the nucleus was not annotated. The dataset included 11,389 annotated TCs, 211,104 annotated TAICs and 5,015 other types of cells.

Second, image patches were extracted centring on the centroid of each segmented nucleus with a size of 54*54 pixels to train the nuclei classifier based on the Xception model [4], which is a state-of-the-art deep neural network architecture. For model training and validation, we randomly separated the annotated nuclei into training (60%), validation (30%) and test (10%) sets. Red circles labelled TCs, while yellow circles labelled TAICs analysed by computational pathology analysis for figure classification and validation (Fig. 1A). In total, 83.6% of the TC nuclei and 87.9% of the TAIC nuclei were correctly classified. Precision, recall and the F1-score were also calculated and demonstrated that the nuclei classifier developed in this study achieved good performance in identifying TCs and TAICs (Fig. 1B). All WSIs were divided into a grid of tiles (1024*1024 pixels), and stain deconvolution, nuclei segmentation and classification were performed for each tile to identify TCs and TAICs.

To identify positive cells, we first performed thresholding on the IHC staining channel to segment all the positive-stained pixels in the tile. For each nucleus, a ring area with a width of 5 pixels was generated at the boundary of the nucleus mask, and the percentage of positive pixels in the defined area was calculated. If the percentage was above a threshold of 5%, the nucleus was classified as positive. Positive cells were labelled with large points in the quantification (Fig. 1A).

The numbers of total TCs, total TAICs, positive TCs and positive TAICs were recorded for each tile and then summed through all the tiles to generate the overall numbers of TCs, TAICs, positive TCs and positive TAICs for the entire WSI. Finally, the positive percentages of TCs and TAICs were calculated for each WSI. The computational pathology pipeline was developed using Python and the TensorFlow library [5].

**DNA Extraction and Real-Time QuantitativePolymerase Chain Reaction**

The plasmatic EBV DNA concentrations were routinely measured before treatment and details were as follow: Peripheral blood (3 ml) were obtained in an EDTA tube and centrifuged at 1600 x g for 15 min for isolation of plasma. Viral DNA was extracted using the QIAamp Blood Kit (Qiagen, Hilden, Germany) and stored at -80°C until further processing. A total of 500 μl plasma samples were used for DNA extraction per column and a final elution volume of 50 μl was used to elute the DNA from the extraction column. A real-time quantitative polymerase chain reaction (PCR) system was developed for plasma EBV DNA detection toward the BamHI-W region of the EBV genome. The sequences of the forward and reverse primers were: 5`-GCCAG AGGTA AGTGG ACTTT-3` and 5`-TACCA CCTCC TCTTC TTGCT-3` respectively. A dual fluorescently-labelled oligomer, 5`-(FAM) CACAC CCAGG CACAC ACTAC ACAT (TAMRA)-3` served as the probe. Amplifications were performed in an Applied Biosystems 7700 Sequence Detector and then analyzed using the Sequence Detection System software (version 1.6.3) developed by Applied Biosystems (Foster City, CA). The plasma EBV DNA concentration was calculated using the following equation: C = Q × (VDNA/VPCR)×(1/VEXT), in which C represents the target concentration in plasma (copies/ml), Q represents the target quantity (copy number) determined by PCR, VDNA represents the total volume of DNA obtained after extraction (typically 50 μl/Qiagen extraction), VPCR represents the volume of DNA solution used for PCR (typically 2 μl) and VEXT represents the volume of plasma extracted (typically 0.5 ml) [6]. In addition, all sample from our study were tested and the results were reported in the Genetic Diagnostic Department of our Cancer Center.

**References**

1. A. C. Ruifrok and D. A. Johnston, “Quantification of histochemical staining by color deconvolution.,” *Analytical and quantitative cytology and histology*, 2001; vol. 23, no. 4, pp. 291-9, Aug.

2. K. He, G. Gkioxari, P. Dollár, and R. Girshick, "Mask r-cnn," in Computer Vision (ICCV), 2017 IEEE International Conference on, pp. 2980-2988: IEEE.

3. https://monuseg.grand-challenge.org/

4. Chollet, F., Xception: Deep Learning with Depthwise Separable Convolutions. p. 1800-1807, 2016.

5. <https://www.tensorflow.org/>

6. Shao JY, Li YH, Gao HY et al. Comparison of plasma Epstein-Barr virus (EBV) DNA levels and serum EBV immunoglobulin A/virus capsid antigen antibody titers in patients withnasopharyngeal carcinoma. Cancer 2004; 100: 1162-1170.

**
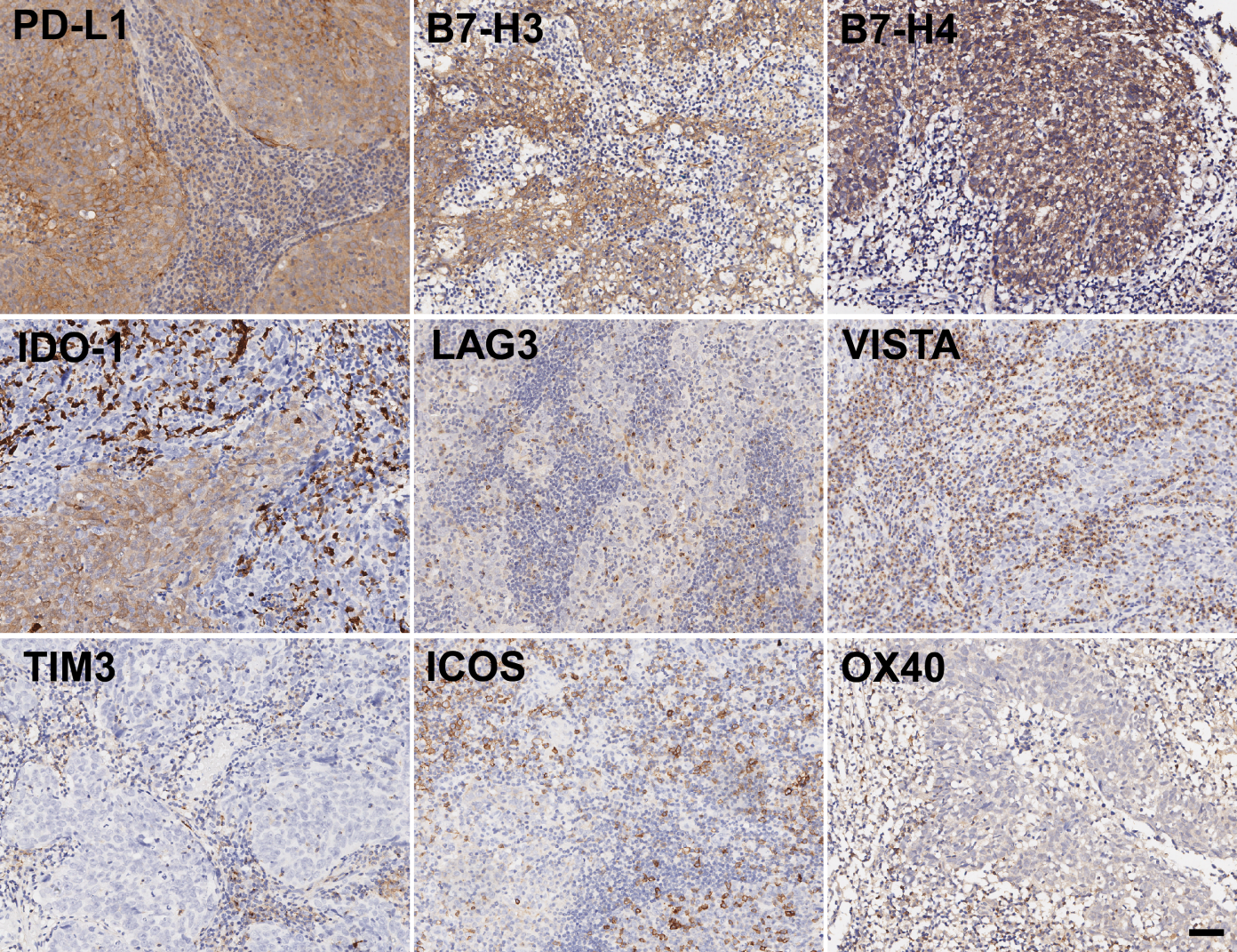
**

**Fig. S1. Representative images of immune checkpoint expression in TCs and TAICs from nasopharyngeal carcinoma determined by immunohistochemistry.**

The immune checkpoints PD-L1, B7-H3, B7-H4, and IDO-1 show positive expression in both TCs and TAICs. LAG3, VISTA, TIM3, ICOS, and OX40 were predominantly expressed in TAICs (original magnification, 200×). The scale bar represents 50 µm. Abbreviations: TCs, tumour cells; TAICs, tumour-associated immune cells.


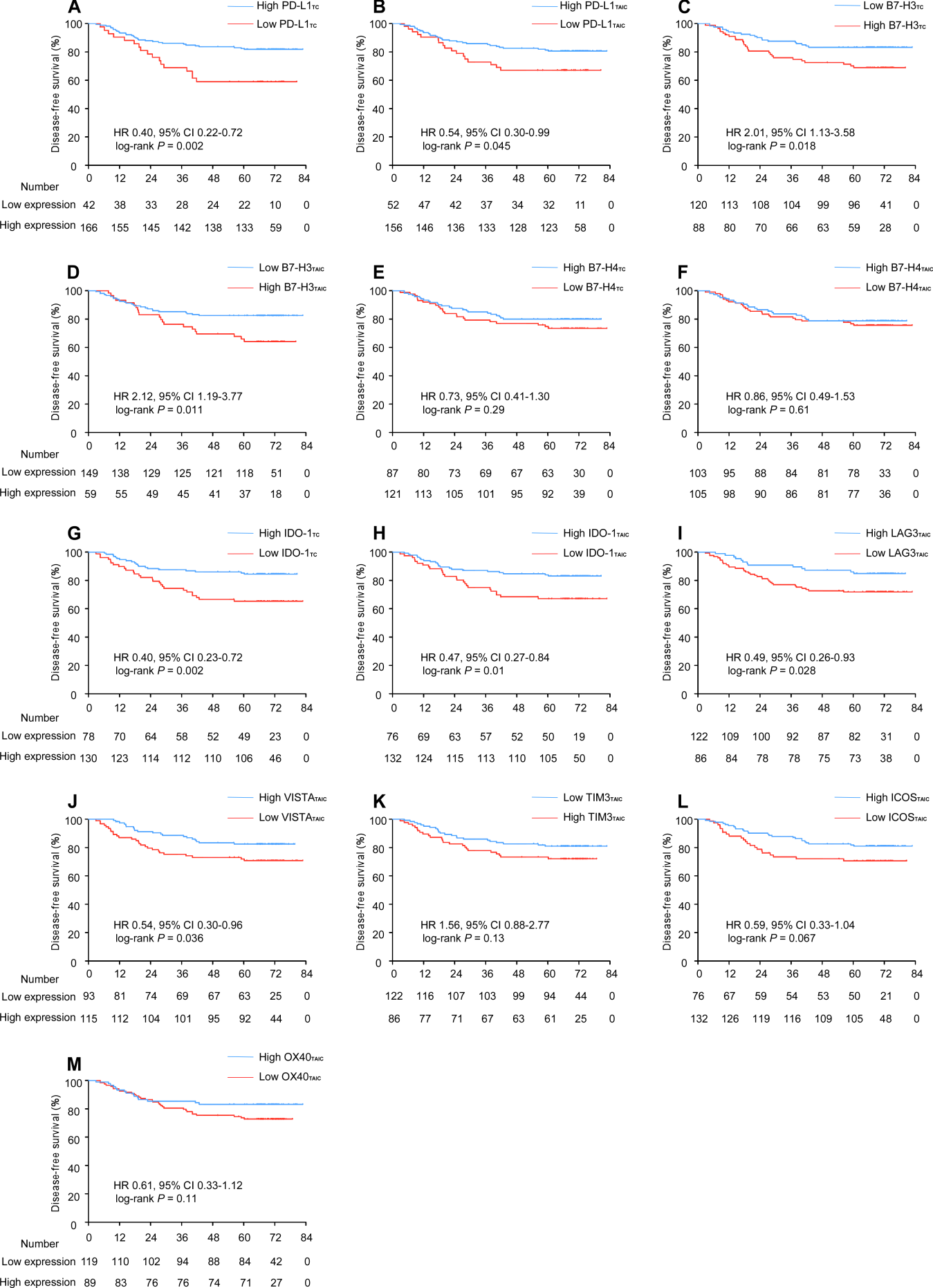


**Fig. S2. Kaplan-Meier curves for disease-free survival according to 13 immune checkpoint features.**

Plots show (A) PD-L1_TC_; (B) PD-L1_TAIC_; (C) B7-H3_TC_; (D) B7-H3_TAIC_; (E) B7-H4_TC_; (F) B7-H4_TAIC_; (G) IDO-1_TC_; (H) IDO-1_TAIC_; (I) LAG3_TAIC_; (J) VISTA_TAIC_; (K) TIM-3_TAIC_; (L) ICOS_TAIC_ and (M) OX40_TAIC_ in the training cohort. Abbreviations: TC, tumour cell; TAIC, tumour-associated immune cell; HR, hazard ratio; CI, confidence interval.


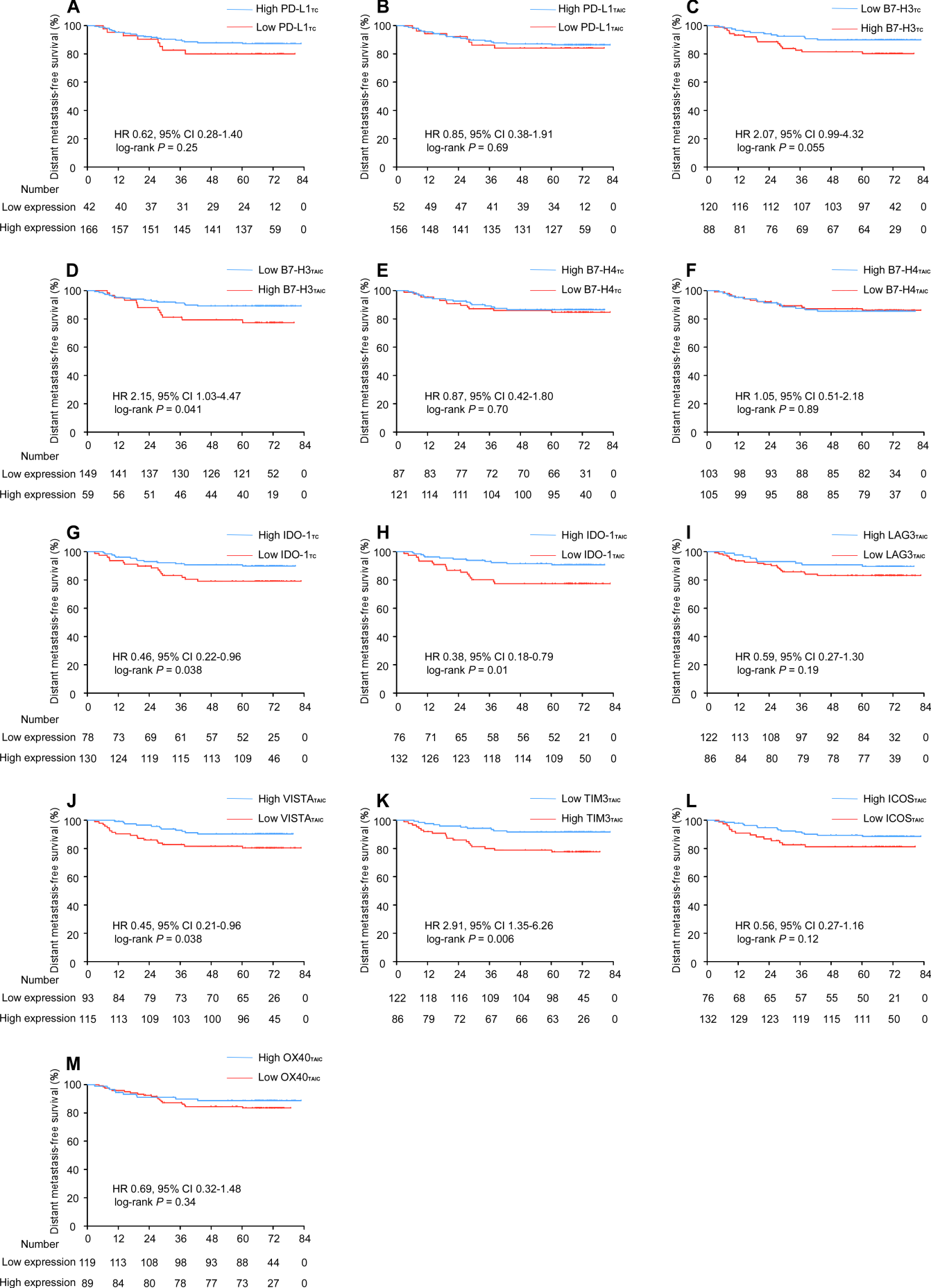


**Fig. S3. Kaplan-Meier curves for distant metastasis-free survival according to 13 immune checkpoint features.**

Plots show (A) PD-L1_TC_; (B) PD-L1_TAIC_; (C) B7-H3_TC_; (D) B7-H3_TAIC_; (E) B7-H4_TC_; (F) B7-H4_TAIC_; (G) IDO-1_TC_; (H) IDO-1_TAIC_; (I) LAG3_TAIC_; (J) VISTA_TAIC_; (K) TIM-3_TAIC_; (L) ICOS_TAIC_ and (M) OX40_TAIC_ in the training cohort. Abbreviations: TC, tumour cell; TAIC, tumour-associated immune cell; HR, hazard ratio; CI, confidence interval.


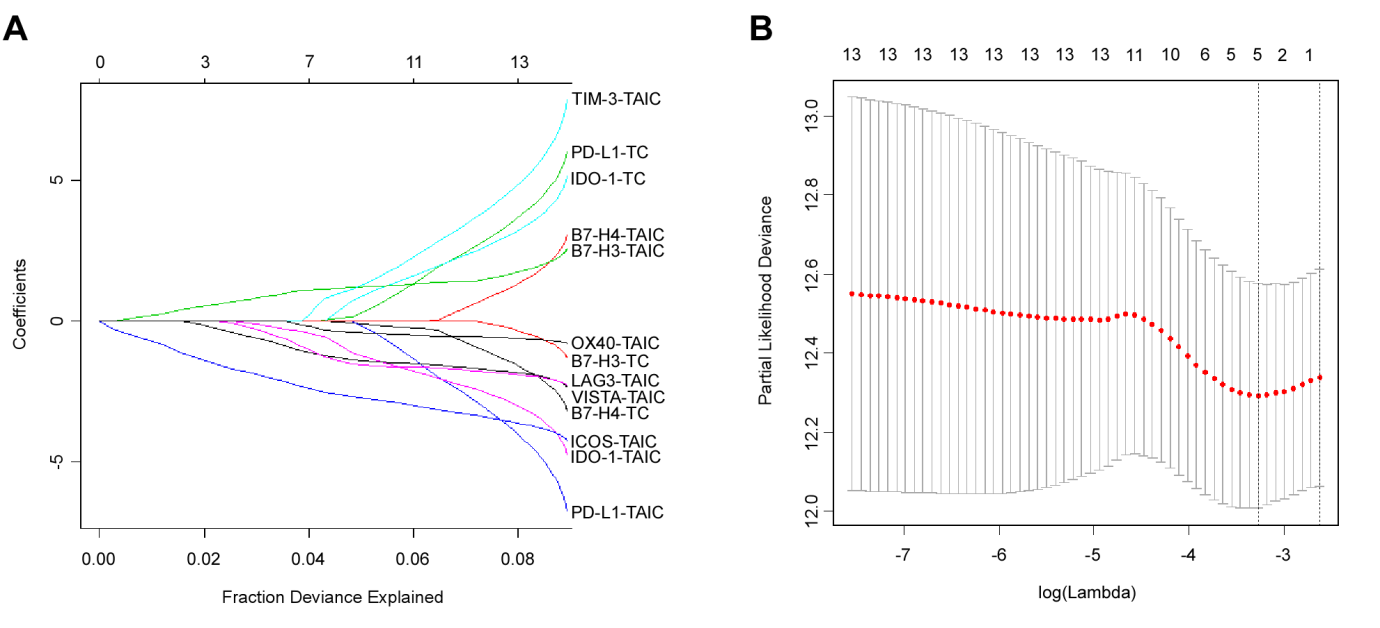


**Fig. S4: Construction of the ICS, a classifier comprising 5 immune checkpoint features.**

(A) LASSO coefficient profiles of 13 immune checkpoint features; (B) ten-time cross-validation for tuning parameter selection in the LASSO model. Abbreviation: ICS, immune checkpoint-based signature.

**
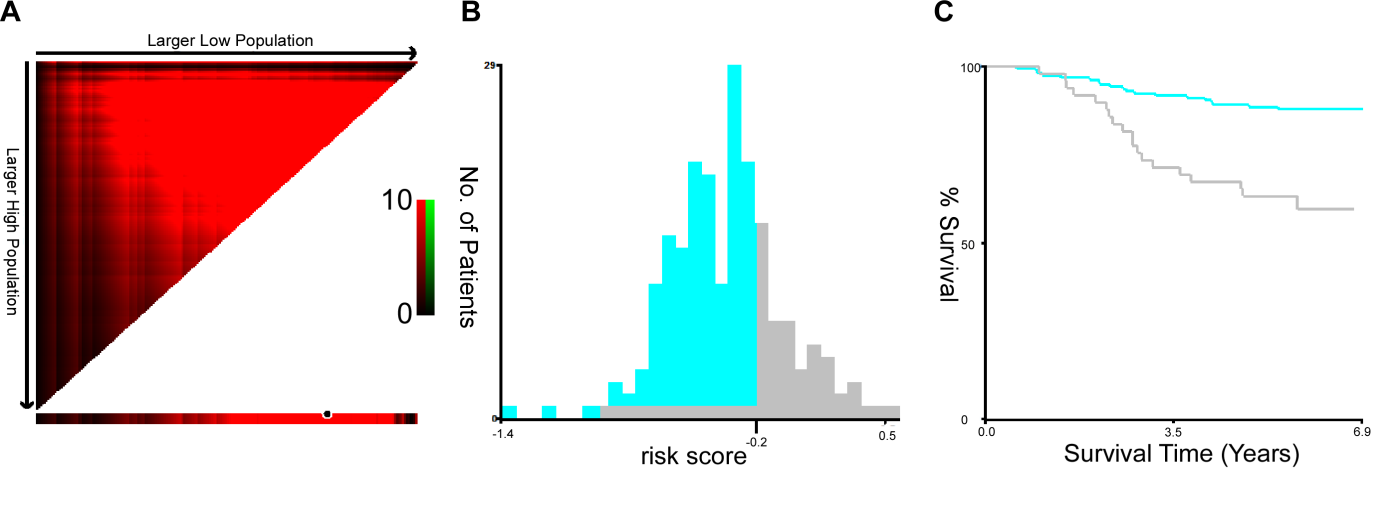
**

**Fig. S5: Determination of the optimum cutoff value for the ICS risk score.**

The optimum cutoff value for the ICS was determined in the training cohort using the X-tile program. The coloration of the plot represents the strength of the association at each division, ranging from low (dark, black) to high (bright, red or green). Red represents an inverse association between the ICS and overall survival, whereas green indicates a direct association. Abbreviation: ICS, immune checkpoint-based signature.
