# Supplementary material for: Development and validation of an immune checkpoint-based signature to predict prognosis in nasopharyngeal carcinoma using computational pathology analysis
Source: J Immunother Cancer. 2019 Nov 13;7:298. doi: 10.1186/s40425-019-0752-4 (PMC6854706; doi:10.1186/s40425-019-0752-4)
Supplement: Supplementary file 2 — Additional file 2: Table S1. Clinicopathological characteristics of the patients in the training and validation cohorts stratified according to the ICS. Table S2. Immune checkpoint co-expression by tumour cells (TCs) in the training cohort. Table S3. Five-year OS, DFS and DMFS estimates for different groups. Table S4. Number of events for different groups. Table S5. Univariate analysis of factors associated with overall survival in the training and validation cohorts. Table S6. Univariate analysis of factors associated with disease-free survival in the training and validation cohorts. Table S7. Univariate analysis of factors associated with distant metastasis-free survival in the training and validation cohorts. Table S8. Multivariable Cox regression analysis of factors associated with survival in the training and validation cohorts. Table S9. Summary of the multivariable analyses of prognostic factors for OS, DFS, DMFS and corresponding risk score in the training set of 208 nasopharyngeal carcinoma patients. [file 40425_2019_752_MOESM2_ESM.docx]

**Table S1. Clinicopathological characteristics of the patients in the training and validation cohorts stratified according to the ICS.**

|  | All | Training cohort (n=208) | | |  | Validation cohort (n=125) | | |
| --- | --- | --- | --- | --- | --- | --- | --- | --- |
|  |  | Low risk | High risk | ***P*** |  | Low risk | High risk | ***P*** |
| **Total population** | 333 (100) | 159 (76.4) | 49 (23.6) |  |  | 70 (46.4) | 55 (36.4) |  |
| **Age** |  |  |  | 0.93 |  |  |  | 0.083 |
| ≤ 45 years | 157 (47.1) | 79 (49.7) | 24 (49.0) |  |  | 35 (50.0) | 19 (34.5) |  |
| > 45 years | 176 (52.9) | 80 (50.3) | 25 (51.0) |  |  | 35 (50.0) | 36 (65.5) |  |
| **Sex** |  |  |  | 0.13 |  |  |  | 0.35 |
| Male | 248 (74.5) | 121 (76.1) | 32 (65.3) |  |  | 51 (72.9) | 44 (80.0) |  |
| Female | 85 (25.5) | 38 (23.9) | 17 (34.7) |  |  | 19 (27.1) | 11 (20.0) |  |
| **WHO pathological type** | |  |  | 0.24 |  |  |  | 0.32 |
| Ⅰ/Ⅱ | 8 (2.4) | 2 (1.3) | 2 (4.1) |  |  | 1 (1.4) | 3 (5.5) |  |
| Ⅲ | 325 (97.6) | 157 (98.7) | 47 (95.9) |  |  | 69 (98.6) | 52 (94.5) |  |
| **T Stage** |  |  |  | 0.12 |  |  |  | 0.057 |
| T1-T2 | 132 (39.6) | 58 (36.5) | 12 (24.5) |  |  | 40 (57.1) | 22 (40.0) |  |
| T3-T4 | 201 (60.4) | 101 (63.5) | 37 (75.5) |  |  | 30 (42.9) | 33 (60.0) |  |
| **N Stage** |  |  |  | 0.56 |  |  |  | 0.78 |
| N0-N1 | 192 (57.7) | 114 (71.7) | 33 (67.3) |  |  | 25 (35.7) | 21 (38.2) |  |
| N2-N3 | 141 (42.3) | 45 (28.3) | 16 (32.7) |  |  | 45 (64.3) | 34 (61.8) |  |
| **TNM Stage** |  |  |  | 0.24 |  |  |  | 0.80 |
| I-II | 80 (24.0) | 46 (28.9) | 10 (20.4) |  |  | 14 (20.0) | 10 (18.2) |  |
| III-IV | 253 (76.0) | 113 (71.1) | 39 (79.6) |  |  | 56 (80.0) | 45 (81.8) |  |
| **Treatment** |  |  |  | 0.56 |  |  |  | 0.58 |
| RT alone | 30 (9.0) | 14 (8.8) | 5 (10.2) |  |  | 7 (10.0) | 4 (7.3) |  |
| CCRT | 140 (42.0) | 57 (35.8) | 19 (38.8) |  |  | 33 (47.1) | 31 (56.4) |  |
| CCRT+AC | 54 (16.3) | 2 (1.3) | 2 (4.1) |  |  | 30 (42.9) | 20 (36.3) |  |
| IC+CCRT | 109 (32.7) | 86 (54.1) | 23 (46.9) |  |  | 0 (0) | 0 (0) |  |
| Dosage of DDP |  |  |  | 0.57 |  |  |  | 0.16 |
| DDP ≤ 200 | 172 (91.0) | 131 (90.3) | 41 (93.2) |  |  | 56 (88.9) | 49 (96.1) |  |
| DDP > 200 | 17 (9.0) | 14 (9.7) | 3 (6.8) |  |  | 7 (11.1) | 2 (3.9) |  |
| **KPS** | |  |  | 0.34 |  |  |  | 0.58 |
| 80 | 8 (2.4) | 3 (1.9) | 2 (4.1) |  |  | 1 (1.4) | 2 (3.6) |  |
| 90 | 325 (97.6) | 156 (98.1) | 47 (95.9) |  |  | 69 (98.6) | 53 (96.4) |  |
| **EBV-DNA load** (**copy/mL)** | |  |  | 0.60 |  |  |  | NA |
| ≤ 2000 | 113 (54.3) | 88 (55.3) | 25 (51.0) |  |  | NA | NA |  |
| > 2000 | 95 (28.5) | 71 (44.7) | 24 (49.0) |  |  | NA | NA |  |
| **Death** |  |  |  | < 0.000 |  |  |  | 0.002 |
| Yes | 75 (22.5) | 19 (11.9) | 19 (38.8) |  |  | 13 (18.6) | 24 (43.6) |  |
| No | 258 (77.5) | 140 (88.1) | 30 (61.2) |  |  | 57 (81.4) | 31 (56.4) |  |
| **Distant metastasis** | |  |  | 0.004 |  |  |  | 0.014 |
| Yes | 55 (16.5) | 16 (10.1) | 13 (26.5) |  |  | 9 (12.9) | 17 (30.9) |  |
| No | 278 (83.5) | 143 (89.9) | 36 (73.5) |  |  | 61 (87.1) | 38 (69.1) |  |
| **Locoregional failure** | |  |  | 0.48 |  |  |  | 0.11 |
| Yes | 50 (15.0) | 14 (8.8) | 6 (12.2) |  |  | 13 (18.6) | 17 (30.9) |  |
| No | 283 (85.0) | 145 (91.2) | 43 (87.8) |  |  | 57 (81.4) | 38 (69.1) |  |
| **Disease progression** | |  |  | 0.002 |  |  |  | 0.002 |
| Yes | 94 (28.2) | 28 (17.6) | 19 (38.8) |  |  | 18 (25.7) | 29 (52.7) |  |
| No | 239 (71.8) | 131 (82.4) | 30 (61.2) |  |  | 52 (74.3) | 26 (47.3) |  |

Abbreviations: ICS, Immune checkpoint-based signature; TNM, Tumour-node-metastasis; IC, induction chemotherapy; CCRT, concomitant chemoradiotherapy; AC, adjuvant chemotherapy; DDP, cisplatin; KPS, Karnofsky; EBV-DNA, Epstein-Barr virus DNA.

**Table S2. Immune checkpoint co-expression by tumour cells (TCs) in the training cohort.**

| Immune checkpoints expressed | NPC, n (%)  (N=208) |
| --- | --- |
| PD-L1, B7-H3, B7-H4, and IDO-1 | 28 (13) |
| PD-L1, B7-H3, and B7-H4 | 16 (8) |
| PD-L1, B7-H3, and IDO-1 | 20 (10) |
| PD-L1, B7-H4, and IDO-1 | 34 (16) |
| B7-H3, B7-H4, and IDO-1 | 4 (2) |
| PD-L1 and B7-H3 | 9 (4) |
| PD-L1 and B7-H4 | 17 (8) |
| PD-L1 and IDO-1 | 26 (13) |
| B7-H3 and B7-H4 | 4 (2) |
| B7-H3 and IDO-1 | 2 (1) |
| B7-H4 and IDO-1 | 13 (6) |
| PD-L1 | 16 (8) |
| B7-H3 | 5 (2) |
| B7-H4 | 5 (2) |
| IDO-1 | 3 (1) |
| No expression | 6 (3) |

Abbreviations: NPC, nasopharyngeal carcinoma.

**Table S3. Five-year OS, DFS and DMFS estimates for different groups.**

| **5-year**  **survival** | Training cohort  (n=208) | | |  | Validation cohort  (n=125) | |
| --- | --- | --- | --- | --- | --- | --- |
|  | Low risk  (n=159) | High risk  (n=49) | |  | Low risk  (n=70) | High risk  (n=55) |
|  |  |  |  |  |  |  |
| **OS** | 88.1 | 61.2 | |  | 81.4 | 56.4 |
| **(95% CI)** | (82.8-93.0) | (49.8-76.8) | |  | (72.1-90.5) | (45.1-71.3) |
| **DFS** | 82.4 | 61.2 | |  | 74.3 | 47.3 |
| **(95% CI)** | (76.3-88.1) | (49.8-76.8) | |  | (64.1-84.5) | (36.0-62.2) |
| **DMFS** | 89.9 | 73.5 | |  | 87.1 | 69.1 |
| **(95% CI)** | (85.1-94.5) | (62.9-87.3) | |  | (78.4-94.8) | (57.6-82.3) |
|  | High EBV-DNA load subgroup  (n=95) | | |  | Low EBV-DNA load subgroup  (n=113) | |
|  | Low risk  (n=71) | | High risk  (n=24) |  | Low risk  (n=88) | High risk  (n=25) |
| **OS** | 83.1 | | 41.7 |  | 92.0 | 80.0 |
| **(95% CI)** | (74.2-91.8) | | (18.5-60.1) |  | (86.2-97.6) | (64.3-95.7) |
| **DFS** | 74.6 | | 41.7 |  | 88.6 | 80.0 |
| **(95% CI)** | (64.4-84.8) | | (21.9-61.5) |  | (81.8-95.2) | (64.3-95.7) |
| **DMFS** | 87.3 | | 54.2 |  | 92.0 | 92.0 |
| **(95% CI)** | (79.3-94.9) | | (30.6-72.6) |  | (86.2-97.6) | (81.4-102.6) |

Abbreviations: OS, overall survival; DFS, disease-free survival; DMFS, distant metastasis-free survival; CI, confidence interval; EBV-DNA, Epstein-Barr virus DNA.

**Table S4. Number of events for different groups.**

| **5-year**  **survival** | Training cohort  (n=208) | |  | Validation cohort  (n=125) | |
| --- | --- | --- | --- | --- | --- |
|  | Low risk  (n=159) | High risk  (n=49) |  | Low risk  (n=70) | High risk  (n=55) |
|  |  |  |  |  |  |
| **Death** | 19 | 19 |  | 13 | 24 |
| **Disease progression** | 28 | 19 |  | 18 | 29 |
| **Distant metastasis** | 16 | 13 |  | 9 | 17 |
|  | High EBV-DNA load subgroup  (n=95) | |  | Low EBV-DNA load subgroup  (n=113) | |
|  | Low risk  (n=71) | High risk  (n=24) |  | Low risk  (n=88) | High risk  (n=25) |
| **Death** | 12 | 14 |  | 7 | 5 |
| **Disease progression** | 18 | 14 |  | 10 | 5 |
| **Distant metastasis** | 9 | 11 |  | 7 | 2 |

Abbreviations: ICS, Immune checkpoint-based signature; EBV DNA, Epstein-Barr virus DNA.

**Table S5. Univariate analysis of factors associated with overall survival in the training and validation cohorts.**

|  | Training cohort (n=208) | |  | Validation cohort (n=125) | |
| --- | --- | --- | --- | --- | --- |
| **Variable** |  | |  |  |  |
|  | HR (95% CI) | ***P*** |  | HR (95% CI) | ***P*** |
| **Overall survival** |  |  |  |  |  |
| ICS (high vs. low) | 3.75 (1.98-7.09) | **< 0.001** |  | 2.58 (1.31-5.07) | **0.006** |
| Age (> 45 years vs. ≤ 45 years) | 1.27 (0.67-2.41) | 0.46 |  | 1.77 (0.89-3.52) | 0.10 |
| Sex (Female vs. Male) | 0.73 (0.33-1.59) | 0.43 |  | 1.06 (0.50-2.24) | 0.88 |
| TNM Stage (III-IV vs. I-II) | 7.30 (1.76-30.3) | **0.006** |  | 10.9 (1.49-79.7) | **0.019** |
| Treatment (IC+CCRT vs. CCRT+AC  vs. CCRT vs. RT alone) | 1.15 (0.86-1.54) | 0.36 |  | 0.87 (0.53-1.43) | 0.58 |
| DDP dosage (Concurrent DDP > 200 vs. ≤ 200) | 0.52 (0.13-2.17) | 0.37 |  | 0.71 (0.17-2.97) | 0.64 |
| KPS (90 vs. 80) | 0.41 (0.10-1.72) | 0.23 |  | 0.31 (0.07-1.28) | 0.11 |
| EBV-DNA (> 2000 vs.≤ 2000) | 2.76 (1.39-5.47) | **0.004** |  | NA | NA |
| LDH (> 245 vs. ≤ 245) | 2.42 (1.11-5.27) | **0.027** |  | NA | NA |
| C-reactive protein (> 3 vs. ≤ 3) | 0.94 (0.48-1.83) | 0.84 |  | NA | NA |
| Haemoglobin (> 120 vs. ≤ 120) | 0.91 (0.28-2.95) | 0.87 |  | NA | NA |

Abbreviations: OS, overall survival; HR, hazard ratio; CI, confidence interval; ICS, Immune checkpoint-based signature; TNM, Tumour-node-metastasis; IC, induction chemotherapy; CCRT, concomitant chemoradiotherapy; AC, adjuvant chemotherapy; DDP, cisplatin; KPS, Karnofsky; EBV-DNA, Epstein-Barr virus DNA; LDH, serum lactate dehydrogenase.

**Table S6. Univariate analysis of factors associated with disease-free survival in the training and validation cohorts.**

|  | Training cohort (n=208) | |  | Validation cohort (n=125) | |
| --- | --- | --- | --- | --- | --- |
| **Variable** |  | |  |  |  |
|  | HR (95% CI) | ***P*** |  | HR (95% CI) | ***P*** |
| **Disease-free survival** |  |  |  |  |  |
| ICS (high vs. low) | 2.51 (1.40-4.50) | **0.002** |  | 2.39 (1.32-4.30) | **0.004** |
| Age (> 45 years vs. ≤ 45 years) | 1.25 (0.70-2.21) | 0.46 |  | 1.36 (0.76-2.45) | 0.30 |
| Sex (Female vs. Male) | 0.74 (0.37-1.50) | 0.41 |  | 0.75 (0.36-1.56) | 0.45 |
| TNM Stage (III-IV vs. I-II) | 4.46 (1.60-12.4) | **0.004** |  | 6.91 (1.67-28.6) | **0.008** |
| Treatment (IC+CCRT vs. CCRT+AC  vs. CCRT vs. RT alone) | 1.28 (0.97-1.68) | 0.08 |  | 0.98 (0.63-1.52) | 0.92 |
| DDP dosage **(**Concurrent DDP > 200 vs. ≤ 200) | 0.43 (0.10-1.76) | 0.24 |  | 0.76 (0.24-2.45) | 0.64 |
| KPS (90 vs. 80) | 0.56 (0.14-2.31) | 0.42 |  | 0.48 (0.12-1.96) | 0.30 |
| EBV-DNA (> 2000 vs.≤ 2000) | 2.78 (1.51-5.14) | **0.001** |  | NA | NA |
| LDH (> 245 vs. ≤ 245) | 2.27 (1.10-4.70) | **0.027** |  | NA | NA |
| C-reactive protein (> 3 vs. ≤ 3) | 0.93 (0.51-1.69) | 0.80 |  | NA | NA |
| Haemoglobin (> 120 vs. ≤ 120) | 0.83 (0.30-2.30) | 0.72 |  | NA | NA |

Abbreviations: DFS, disease-free survival; HR, hazard ratio; CI, confidence interval; ICS, Immune checkpoint-based signature; TNM, Tumour-node-metastasis; IC, induction chemotherapy; CCRT, concomitant chemoradiotherapy; AC, adjuvant chemotherapy; DDP, cisplatin; KPS, Karnofsky; EBV-DNA, Epstein-Barr virus DNA; LDH, serum lactate dehydrogenase.

**Table S7. Univariate analysis of factors associated with distant metastasis-free survival in the training and validation cohorts.**

|  | Training cohort (n=208) | |  | Validation cohort (n=125) | |
| --- | --- | --- | --- | --- | --- |
| **Variable** |  | |  |  |  |
|  | HR (95% CI) | ***P*** |  | HR (95% CI) | ***P*** |
| **Distant metastasis-free survival** |  |  |  |  |  |
| ICS (high vs. low) | 2.93 (1.40-6.09) | **0.004** |  | 2.55 (1.13-5.73) | **0.024** |
| Age (> 45 years vs. ≤ 45 years) | 1.08 (0.52-2.23) | 0.84 |  | 1.33 (0.60-2.92) | 0.49 |
| Sex (Female vs. Male) | 0.87 (0.37-2.04) | 0.75 |  | 0.56 (0.19-1.63) | 0.29 |
| TNM Stage (III-IV vs. I-II) | 5.49 (1.31-23.1) | **0.02** |  | 7.52 (1.02-55.7) | **0.048** |
| Treatment (IC+CCRT vs. CCRT+AC  vs. CCRT vs. RT alone) | 1.45 (0.99-2.09) | 0.051 |  | 0.68 (0.38-1.21) | 0.19 |
| DDP dosage **(**Concurrent DDP > 200 vs. ≤ 200) | 0.38 (0.051-2.77) | 0.34 |  | 1.03 (0.24-4.40) | 0.96 |
| KPS (90 vs. 80) | 0.33 (0.08-1.37) | 0.13 |  | 0.54 (0.07-4.00) | 0.55 |
| EBV-DNA (> 2000 vs.≤ 2000) | 2.82 (1.28-6.19) | **0.01** |  | NA | NA |
| LDH (> 245 vs. ≤ 245) | 2.89 (1.23-6.77) | **0.014** |  | NA | NA |
| C-reactive protein (> 3 vs. ≤ 3) | 0.81 (0.37-1.78) | 0.60 |  | NA | NA |
| Haemoglobin (> 120 vs. ≤ 120) | 0.67 (0.20-2.20) | 0.51 |  | NA | NA |

Abbreviations: DMFS, distant metastasis-free survival; HR, hazard ratio; CI, confidence interval; ICS, Immune checkpoint-based signature; TNM, Tumour-node-metastasis; IC, induction chemotherapy; CCRT, concomitant chemoradiotherapy; AC, adjuvant chemotherapy; DDP, cisplatin; KPS, Karnofsky; EBV-DNA, Epstein-Barr virus DNA; LDH, serum lactate dehydrogenase.

**Table S8. Multivariable Cox regression analysis of factors associated with survival in the training and validation cohorts.**

|  | Training cohort (n=208) | |  | Validation cohort (n=125) | |
| --- | --- | --- | --- | --- | --- |
| **Variable** |  | |  |  |  |
|  | HR (95% CI) | ***P*** |  | HR (95% CI) | ***P*** |
| **overall survival** |  |  |  |  |  |
| ICS (high vs. low) | 3.62 (1.91-6.87) | < 0.001 |  | 2.59 (1.32-5.10) | 0.006 |
| TNM Stage (III-IV vs. I-II) | 5.64 (1.34-23.7) | 0.018 |  | 11.0 (1.50-80.2) | 0.018 |
| EBV-DNA (> 2000 vs.≤ 2000) | 2.37 (1.19-4.72) | 0.014 |  | NA | NA |
| **disease-free survival** |  |  |  |  |  |
| ICS (high vs. low) | 2.43 (1.35-4.35) | 0.003 |  | 2.38 (1.32-4.30) | 0.004 |
| TNM Stage (III-IV vs. I-II) | 3.51 (1.25-9.89) | 0.018 |  | 6.89 (1.67-28.5) | 0.008 |
| EBV-DNA (> 2000 vs.≤ 2000) | 2.39 (1.29-4.45) | 0.006 |  | NA | NA |
| **distant metastasis-free survival** |  |  |  |  |  |
| ICS (high vs. low) | 2.77 (1.33-5.77) | 0.007 |  | 2.55 (1.13-5.72) | 0.024 |
| TNM Stage (III-IV vs. I-II) | 4.25 (1.00-18.1) | 0.050 |  | 7.49 (1.01-55.5) | 0.049 |
| EBV-DNA (> 2000 vs.≤ 2000) | 2.41 (1.09-5.34) | 0.029 |  | NA | NA |

Abbreviations: HR, hazard ratio; CI, confidence interval; ICS, Immune checkpoint-based signature; TNM, Tumour-node-metastasis; EBV-DNA, Epstein-Barr virus DNA.

**Table S9. Summary of the multivariable analyses of prognostic factors for OS, DFS, DMFS and corresponding risk score in the training set of 208 nasopharyngeal carcinoma patients.**

| **Variable** | **β Coefficient** | **HR** | **95% CI for HR** | ***P-*value** | **Risk sore** |
| --- | --- | --- | --- | --- | --- |
| **overall survival** | | | | | |
| **ICS** |  |  |  |  |  |
| Low risk | 1 | 1 |  |  | 0 |
| High risk | 1.29 | 3.62 | 1.91-6.87 | < 0.001 | 1 |
| **TNM Stage** |  |  |  |  |  |
| I-II | 1 | 1 |  |  | 0 |
| III-IV | 1.73 | 5.64 | 1.34-23.7 | 0.018 | 1 |
| **disease-free survival** | | | | | |
| **ICS** |  |  |  |  |  |
| Low risk | 1 | 1 |  |  | 0 |
| High risk | 0.89 | 2.43 | 1.35-4.35 | 0.003 | 1 |
| **TNM Stage** |  |  |  |  |  |
| I-II | 1 | 1 |  |  | 0 |
| III-IV | 1.26 | 3.51 | 1.25-9.89 | 0.018 | 1 |
| **distant metastasis-free survival** | | | | | |
| **ICS** |  |  |  |  |  |
| Low risk | 1 | 1 |  |  | 0 |
| High risk | 1.07 | 2.77 | 1.33-5.77 | 0.007 | 1 |
| **TNM Stage** |  |  |  |  |  |
| I-II | 1 | 1 |  |  | 0 |
| III-IV | 1.47 | 4.25 | 1.00-18.1 | 0.050 | 1 |

Abbreviations: OS, overall survival; DFS, disease-free survival; DMFS, distant metastasis-free survival; HR, hazard ratio; CI, confidence interval; ICS, Immune checkpoint-based signature; TNM, Tumour-node-metastasis.
